# Using Active Engagement to Support Dementia Caregivers: Results from the Enhancing Active Caregiver Training (EnACT) Study

**DOI:** 10.1002/alz70858_105734

**Published:** 2025-12-26

**Authors:** Jacqueline Eaton, Eli Iacob, Amber Dayley, Moroni Fernandez Cajavilca, Sarah Neller, Julene K. Johnson, Rebecca Utz, Lee Ellington

**Affiliations:** ^1^ University of Utah, Salt Lake City, UT, USA; ^2^ New York University, New York, NY, USA; ^3^ University of Tennessee, Knoxville, Knoxville, TN, USA; ^4^ University of California San Francisco, San Francisco, CA, USA

## Abstract

**Background:**

Actively engaging caregivers in applying knowledge and skills through training and support groups improves caregiver outcomes. However, the optimization and systematic testing of active engagement in these settings are lacking. The Enhancing Active Caregiver Training (EnACT) intervention aims to foster active engagement through arts‐based, multisensory experiences, helping caregivers manage common dementia‐related behavioral symptoms. EnACT uses caregiver‐informed vignettes and imagination exercises that enhance active engagement by practicing caregiving skills and reactions in settings that allow for trial and error, better preparing caregivers for challenging situations.

We examined the effects of EnACT on two proximal outcomes (capacity to adapt and appraisal of caregiving demands), the distal outcomes of perceived stress and caregiver well‐being (positive aspects of caregiving and caregiver burden), and three proposed mechanisms of action (increased preparation, increased multisensory detail, and decreased discrepancy).

**Methods:**

Thirty caregivers of persons living with dementia were randomly assigned to the EnACT intervention (*n* = 15) or waitlist control (WLC) (*n* = 15) groups. All participants completed baseline, pretreatment (2 weeks), 3 sessions of ENACT over 6 weeks, and a post‐treatment. Data was gathered at six timepoints to assess proximal and distal outcomes, and mechanisms of action. We used linear models conditioning on baseline values to examine the impact of the intervention on our outcomes. Furthermore, we included data of the intervention effect on the WLC group to leverage their exposure when estimating our parameters.

**Results:**

Participants in both groups had high retention rates with 97% completing all 6 questionnaires. Exploratory analysis found that the EnACT intervention led to small effect size (Cohen's d 0.2 to 0.3) improvements in both the intervention and WLC groups in perceived stress, two appraisal of caregiving demand subscales (burden, satisfaction), positive aspects of caregiving, and preparation. Additional variables did not show change.

**Conclusions:**

Initial results demonstrate that EnACT has the potential of reducing perceived stress, improving preparation and positive aspects of caregiving, while steadying burden across time. Time in session and number of sessions may influence outcomes, suggesting a need for refinement in intervention dose. Data from this pilot is being used to prepare a fully powered randomized controlled study.

